# Advances in Therapeutic Melanoma Vaccines (2010–2025)

**DOI:** 10.3390/vaccines14080682

**Published:** 2026-08-07

**Authors:** Michael Y. Bian, Reagan Stevens, Ankit Mangla, Devarati Mitra, Nicholas G. Zaorsky, Jeremy S. Bordeaux, Luke D. Rothermel

**Affiliations:** 1 Case Western Reserve University School of Medicine, Cleveland, OH 44106, USA; 2 Department of Surgery, Division of Surgical Oncology, University Hospitals Cleveland Medical Center, Cleveland, OH 44106, USA; sarah.stevens@uhhospitals.org; 3 Case Comprehensive Cancer Center, Cleveland, OH 44106, USA; 4 Department of Hematology and Oncology, University Hospitals Seidman Cancer Center, Cleveland, OH 44106, USA; 5 Department of Radiation Oncology, The University of Texas MD Anderson Cancer Center, Houston, TX 77030, USA; 6 Department of Radiation Oncology, University Hospitals Cleveland Medical Center, Cleveland, OH 44106, USA; 7 Department of Dermatology, University Hospitals Cleveland Medical Center, Cleveland, OH 44106, USA

**Keywords:** melanoma, advanced melanoma, cancer vaccine, immunotherapy, vaccine mechanism, neoantigen, mRNA vaccine, dendritic cell vaccine

## Abstract

**Background/Objectives**: Immune checkpoint inhibitors (ICIs) and other systemic therapies can extend the survival of many patients with advanced melanoma, renewing interest in complementary strategies for unresectable or metastatic disease. Therapeutic cancer vaccines represent a possible approach. Recent evidence suggests commercially available mRNA vaccines can sensitize tumors to ICIs, challenging prior assumptions about neoantigen presentation. Despite renewed public interest in personalized cancer vaccine development, the proliferation of narrative and scoping reviews has not been matched by systematic mechanistic synthesis—platforms investigated across the 2010s and 2020s have not been rigorously compared within a single article. Accordingly, this review focuses on major vaccine platforms and proposes a comparative framework for understanding therapeutic melanoma vaccine development and immunologic rationale. **Methods**: Melanoma vaccine clinical trials listed on ClinicalTrials.gov were identified from 2010 to 2025. Eligible studies were screened and included in a qualitative review evaluating trial characteristics, vaccine platforms, study design, and reported outcomes. **Results**: There is one active Phase III clinical trial for melanoma vaccines in the US. Key principles of melanoma vaccinology include adjuvant, antigen, and delivery platform selection. From 2010 to 2025, four vaccine candidates advanced to Phase III trials, two provided Phase III results for peer review, one trial is ongoing, and no vaccines have been FDA-approved. Inconsistent reporting of outcomes and a lack of published results limited comparability across studies, precluding formal meta-analysis. **Conclusions**: Therapeutic melanoma vaccine options remain limited by translational challenges in study design and reporting gaps. This review synthesizes contemporary clinical trial activity and immunologic principles to inform future research.

## 1. Introduction

Melanoma is the fifth most common cancer in the US, with cutaneous melanoma accounting for ~94% of cases and 104,960 projected new cases in 2025 [1,2]. As immunotherapies have extended the survival of patients with advanced cutaneous melanoma, recurrence remains a substantial concern. Population-level data suggest recurrence in ~10–25% of patients overall, with cumulative incidence rising sharply by stage [3,4,5]. Five-year recurrence rates range from 1.6% for AJCC8 stage IA disease to approximately 35% for stage IIB/IIC/IIIB, exceeding 40% in stage IIIC–IV disease. Most recurrences in higher-stage disease occur in the first 2 years, with >50% of first recurrences presenting as distant metastases carrying a much worse prognosis than locoregional disease alone [3,6].

Immune checkpoint inhibitors have transformed melanoma management; however, durable disease control remains limited across both adjuvant and metastatic settings. Real-world data indicate recurrence in ~50% of patients receiving adjuvant monotherapy by 5 years, with a median time to recurrence of 6.2 months [7]. Adjuvant treatment may offer a relapse-free survival benefit but no overall survival benefit [8]. In the metastatic setting, combination immunotherapy has become widely adopted, with nivolumab/ipilimumab extending median overall survival to ~71.9 months (5-year OS 95%) compared to 36.9 months for nivolumab alone (5-year OS 96%) and 19.9 months for ipilimumab alone (5-year OS 90%), although the study was not designed to compare combination vs. anti-PD1 monotherapy [9]. Resistance to treatment is strongly linked to primary refractory disease or acquired resistance [6,10,11,12].

Current consensus guidelines recommend neoadjuvant immunotherapy for clinically detectable resectable stage III disease, with adjuvant therapy and surgical excision for high-risk resectable disease, and systemic therapy with metastasis-directed treatment and/or observation for unresectable disease [13,14,15,16]. Agents have emerged in this treatment refractory space. Tumor-infiltrating lymphocyte therapy (Lifileucel) has demonstrated a 31.4% objective response rate and median overall survival of 13.9 months; among responders, the median duration of response was 36.5 months [17,18]. This landscape necessitates approaches to identify and target drivers of treatment resistance in melanoma.

Therapeutic cancer vaccines represent one such strategy. From June 2010 to September 2025, 145 trials for therapeutic melanoma vaccines were registered and initiated on ClinicalTrials.gov. The search criteria and PRISMA guidelines are available as Supplementary Material (Section S1, Table S1, Figure S1). Recent evidence suggests commercially available mRNA vaccines can promote anti-tumor responses to tumor antigens, challenging prior assumptions about neoantigen mRNA presentation mechanisms [19]. Positive results from the KEYNOTE-942 trial, demonstrated a 49% reduction in the risk of recurrence or death when a personalized mRNA vaccine was combined with anti-PD1 monotherapy, compared to anti-PD1 monotherapy alone [20,21]. With renewed public and sustained scientific interest in vaccine development, recent reviews have largely focused on survival outcomes or neoantigen-specific mechanistic discussions, without a systematic synthesis that bridges these emerging mechanistic insights with earlier investigative platforms [22,23,24,25,26,27]. This review focuses on major vaccine platforms of the past 15 years and proposes a comparative framework for understanding therapeutic melanoma vaccine development and immunologic rationale.

Our review found limited published data, substantial heterogeneity in trial design and outcomes, and inconsistent reporting, which precludes meta-analysis and obscures meaningful conclusions. Much of the relevant immunology also remains siloed from clinical disciplines with limited visibility of the translational landscape.

The purpose of this review article is to introduce the key concepts and trial designs used by therapeutic melanoma vaccines in the management of patients with advanced stage disease. Section 2 details advances in vaccine design since 2010 and future directions. Section 3, Section 4, Section 5 and Section 6 provide an overview of immunologic principles to understand Section 2. Section 7 contains active trials. Section 8 provides a critique of the current state of clinical trial reporting. This article also aims to support clinical immunologists, radiation oncologists, dermatologists, and surgical oncologists by highlighting strategies in Phase II/III trials and potential innovations in the treatment of advanced melanoma.

## 2. What Advances Have Been Made Since 2010?

Before 2010, therapeutic melanoma vaccines had accumulated decades of largely negative Phase III results despite consistent immunogenicity and encouraging early-phase signals. Multiple vaccine strategies predominantly targeted non-mutated, shared tumor-associated antigens (TAAs) or whole-cell tumor preparations, which consistently induced antigen-specific immune responses but failed to improve survival in randomized clinical trials. High-profile examples include the allogeneic whole-cell vaccine Canvaxin, the multi-epitope peptide vaccine (gp100, MART-1, tyrosinase) evaluated in ECOG 1696, the recombinant MAGE-A3 protein antigen in the DERMA trial, dendritic cell vaccination compared with dacarbazine, and GM2 ganglioside vaccine in EORTC 18961, all of which failed to demonstrate superiority over standard-of-care therapy [28,29,30,31,32].

The year 2010 was pivotal for tumor immunotherapy with two landmark randomized trials. First, the IMPACT trial established sipuleucel-T as the first FDA-approved therapeutic cancer vaccine, based on a 22% relative reduction in risk of death and 4.1-month median survival benefit in metastatic castration-resistant prostate cancer. The mechanism of sipuleucel-T involves the ex vivo activation of autologous antigen-presenting cells, which fundamentally differs from aforementioned melanoma vaccine trials [33]. That same year, Hodi et al. reported the first randomized Phase III trial of ipilimumab, which demonstrated an overall survival benefit in metastatic melanoma, thus establishing immune checkpoint blockage as an effective therapeutic strategy. Of note, the gp100 peptide vaccine used as a comparator in the ipilimumab trial produced limited benefit, and the ipilimumab/gp100 vaccine combination failed to improve outcomes beyond checkpoint inhibition alone [34]. These results provided further evidence that non-specific TAAs offered little value in enhancing T-cell activation. In other words, priming T cells against tumor antigens has little efficacy if native T cells experience suppression within the tumor microenvironment.

In 2015, the FDA approved talimogene laherparepvec (T-VEC) as the first FDA-approved oncolytic virus for melanoma [35,36]. T-VEC is a modified herpes simplex virus encoding GM-CSF that converts immunologically cold tumors into hot tumors. In a Phase III trial of unresectable stage IIIB–IV melanoma, T-VEC for locoregional disease control achieved durable responses in 16.3% of patients compared to 2.1% with GM-CSF, and improved median overall survival (23.3 vs. 18.9 months). Combination studies, such as the Phase II T-VEC plus ipilimumab trial, demonstrated improved durable response rates compared to ipilimumab alone (33.7% vs. 13.0%) [37]. Early-phase studies combining T-VEC with pembrolizumab demonstrated high response rates and T-cell infiltration; however, the Phase III MASTERKEY-265 trial did not significantly improve progression-free or overall survival compared to pembrolizumab monotherapy [38,39].

Since 2010, therapeutic melanoma vaccine development has undergone a major conceptual and philosophical shift from generic shared antigens toward personalized, multi-agent strategies. Advances in high-throughput next-generation sequencing and machine learning HLA-binding prediction algorithms now enable the rapid identification of non-synonymous somatic mutations (neoantigens), which potentially bypass thymic negative selective and could induce high-affinity CD8^+^ and CD4^+^ T-cell responses without triggering autoimmune toxicity or central tolerance. Innovations in vaccine platforms, including lipid nanoparticle (LNP)-encapsulated mRNA, have overcome many of the manufacturing and scalability limitations of earlier vaccine approaches [40]. These platforms can efficiently deliver multi-epitope personalized constructs directly to professional antigen-presenting cells. Therapeutic vaccines are no longer developed as standalone treatments but as components of rational combination immunotherapy. Personalized neoantigen vaccines expand the repertoire of tumor-specific T-cell clones, whereas immune checkpoint inhibitors potentiate their function within the tumor microenvironment.

The current rationale for therapeutic cancer vaccines has translated into encouraging clinical outcomes. For instance, in the randomized Phase II KEYNOTE-942 trial, the individualized intismeran autogene (formerly known as mRNA-4157/V940) vaccine combined with pembrolizumab reduced the risk of recurrence or death by 44% compared to pembrolizumab alone in patients with high-risk resected melanoma (HR, 0.56), providing the first randomized evidence that personalized cancer vaccines can improve clinically meaningful outcomes and warrant ongoing Phase III evaluation. Collectively, these advances define a new era of melanoma vaccine development characterized by precision antigen selection, scalable vaccine manufacturing, and biologically rational combination strategies that directly address many of the limitations that constrained earlier generations of therapeutic vaccines.

## 3. Introduction to Vaccine Immunology

In the 1790s, Edward Jenner demonstrated that controlled immune stimulation could protect against smallpox disease, laying the foundations for modern immunology [41]. By the late 19th century, the concept of vaccination expanded beyond prevention to include therapeutic applications. The first immune stimulatory therapy by William Coley involved a mixture of heat-killed *Streptococcus pyogenes* and *Serratia marcesans* bacteria—later termed “Coley’s toxins”—to treat sarcoma patients by triggering immune responses that caused tumor regression [42]. Coley reportedly treated almost 1000 patients with his concoction, yet other physicians struggled to reproduce his results due to a combination of poor follow-up, 13 different preparations, and different injection sites [43,44,45]. The modern understanding is that Coley’s approach worked by eliciting a strong innate immune response with subsequent activation of adaptive immunity, including T-cell-mediated responses [46].

The immune response is broadly divided into innate and adaptive arms. Innate immunity is the nonspecific, rapid response to antigens that involves neutrophils, macrophages, dendritic cells, natural killer (NK) cells, complement, physical barriers, and secreted enzymes. Adaptive immunity is a highly specific response developed through exposure or vaccinations that involves T and B cells and circulating antibodies. Central to adaptive immunity is antigen presentation: major histocompatibility complex (MHC) class I presents endogenous antigens (viral or cytosolic proteins) to CD8^+^ cytotoxic T cells, while MHC class II molecules present exogenous antigens (e.g., bacterial proteins) to CD4^+^ helper T cells that coordinate cellular and humoral immunity [47].

Since Coley’s early experiments, a fundamental question in cancer vaccine development has been how to overcome immune tolerance and engage the innate and adaptive immune system to elicit T-cell responses capable of mediating tumor regression [48]. The three core pillars of melanoma vaccinology include antigen selection, adjuvant design to enhance immune activation, and optimized delivery platforms to facilitate efficient antigen presentation [49,50,51]. Melanoma vaccines may be personalized or off-the-shelf [52].

## 4. Antitumor Vaccine Mechanisms

Successful T-cell-mediated antitumor immunity depends on several interrelated elements of vaccine design. These include (1) antigen delivery in high quantities and high quality; (2) efficient capture, processing, and presentation of tumor antigens by antigen-presenting cells (APCs), such as dendritic cells (DCs); (3) activation of CD4^+^ and CD8^+^ T cells; (4) T-cell infiltration of the tumor microenvironment and effective antigen/MHC T-cell receptor-mediated destruction of tumor cells; and (5) maintenance of the immune response driven by chemokine/cytokine gradients [53].

Adaptive T-cell immunity is initiated within the secondary lymphoid organs. Primary lymphoid organs include the bone marrow and thymus, which produce immune cells and allow for B/T-cell maturation. Secondary lymphoid organs include the spleen, lymph nodes, tonsils, adenoids, appendix, and Peyer’s patches, which provide sites for immune cells to interact with antigens [54,55]. Secondary lymphoid organs facilitate nonspecific filtration of macrophages, circulation and interaction of B and T cells, and adaptive immune response activation through associated chemokines, cytokines, and intracellular signaling cascades [56,57,58].

**Figure 1:**
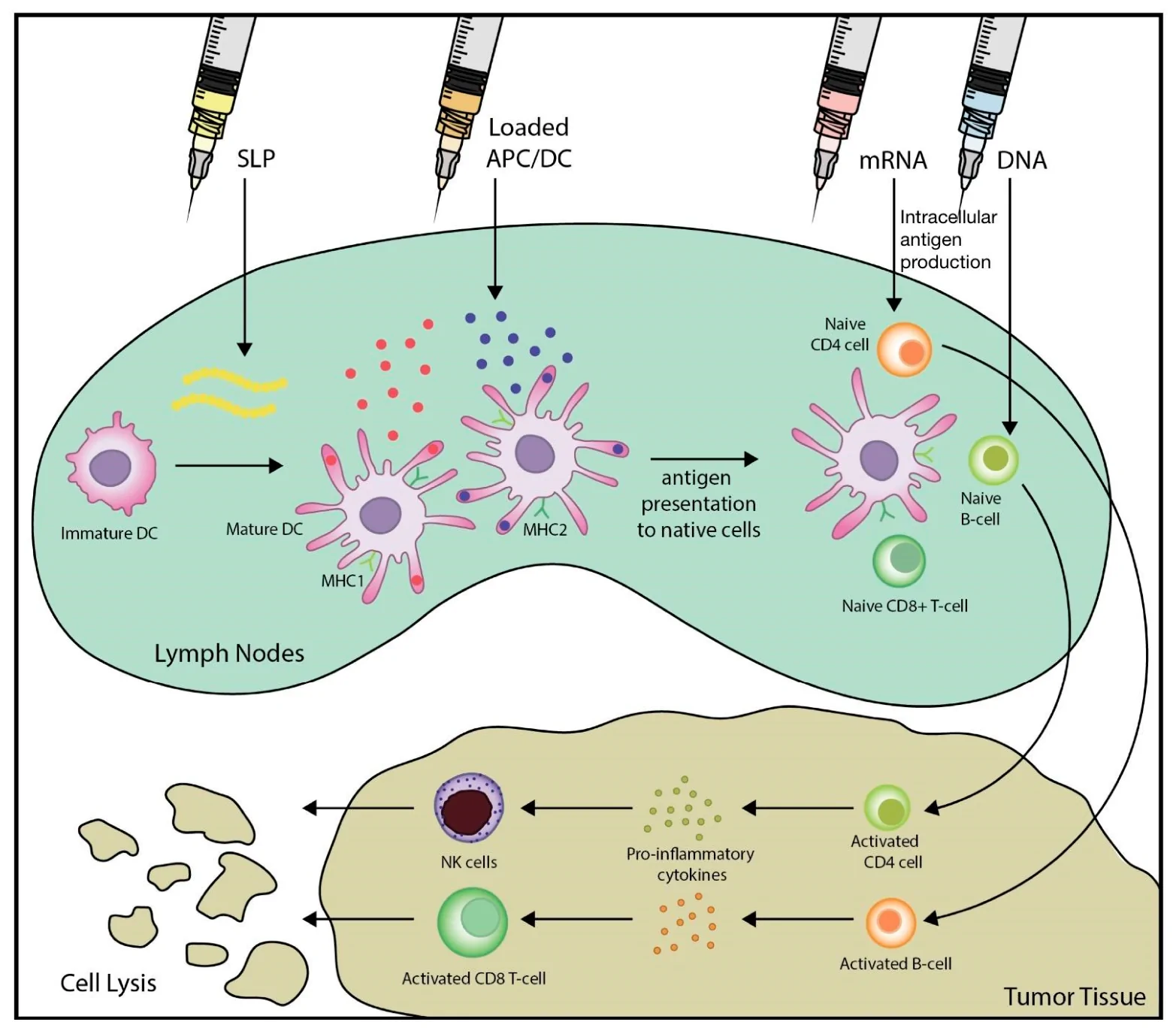
Mechanism of adaptive immune response to a vaccine. Illustration of immune system activation by major vaccine platforms. Arrows show cascade of immune activation and where vaccine platforms exert effects. In lymph nodes and other secondary lymphoid organs, antigens from SLPs or antigens (from loaded DCs) are ingested by immature dendritic cells and presented via MHC I and II to naive CD8 T cells, CD4 T cells, and B cells. Mature immune cells migrate to the tumor site, where CD4^+^ T cells and B cells secrete pro-inflammatory cytokines, while CD8^+^ T cells and NK cells promote tumor cell lysis.

Figure 1 illustrates immune priming within the lymph node. Antigens from synthetic long peptides (SLPs), vaccine-derived antigens, or ex vivo loaded DCs are ingested by immature dendritic cells, accompanied by protein processing, migration, maturation, and presentation to stimulate antigen-specific lymphocyte clones via MHC class I and class II pathways [59]. Naïve CD8^+^ T cells, CD4^+^ T cells, and B cells are activated and undergo clonal expansion. Mature effector cells subsequently migrate to the tumor microenvironment, where CD4^+^ T cells and B cells secrete pro-inflammatory cytokines, while CD8^+^ T cells and natural killer (NK) cells mediate direct tumor cell lysis [60,61,62,63,64,65,66].

**Figure 2:**
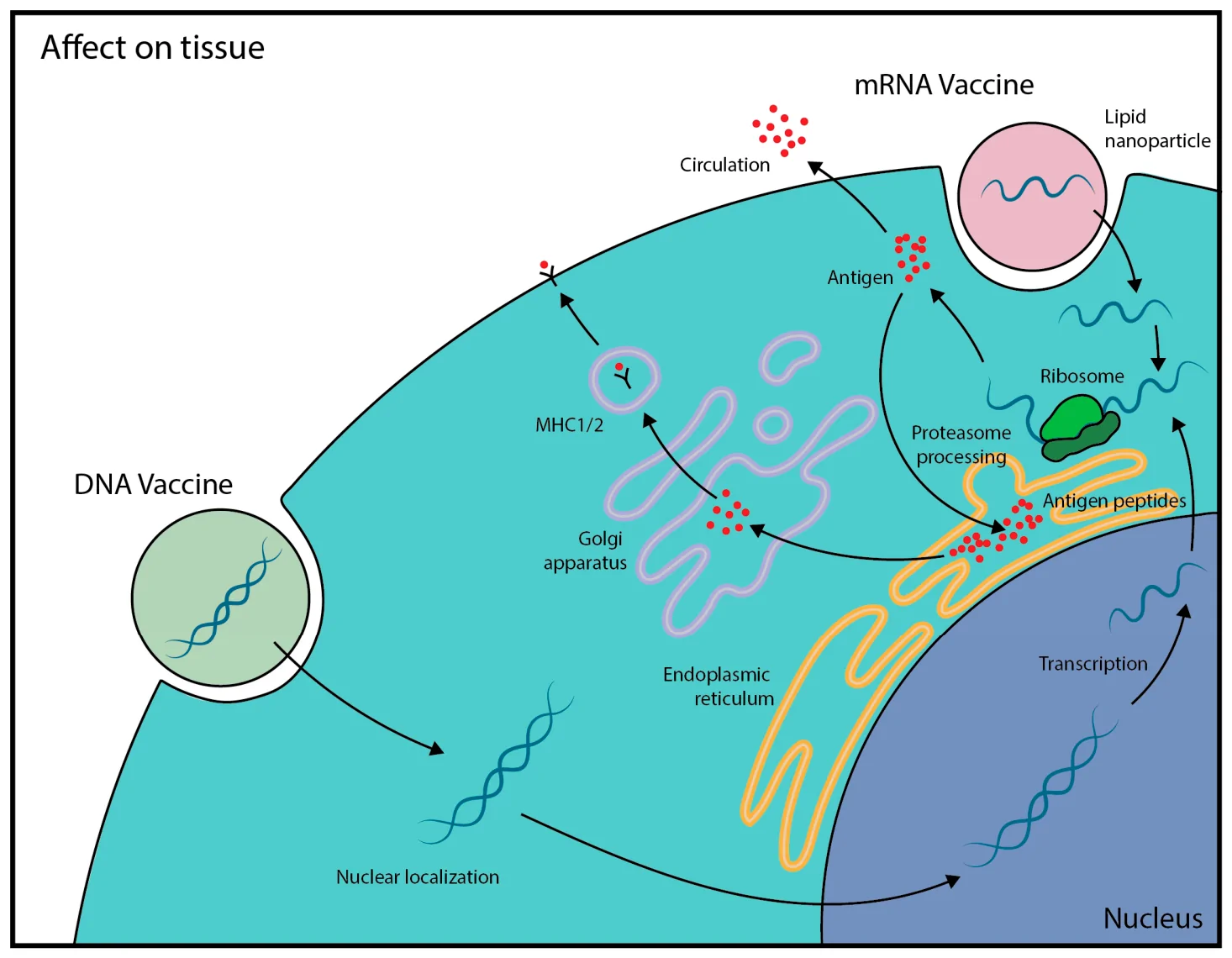
Mechanism of nucleic acid vaccine approaches. Illustration of intracellular processing of DNA and mRNA vaccines following tissue administration. Arrows depict intracellular trafficking of nucleic acids and antigen processing pathways. DNA vaccines (left) enter cell and translocate to nucleus, which produces mRNA that is exported to cytoplasm. mRNA vaccines (Right) remain in the cytoplasm and are translated by ribosomes. Both vaccine types produce tumor-specific antigens that are processed in the endoplasmic reticulum and Golgi apparatus into antigenic peptides. Peptides are then presented on MHC class I and II molecules that initiate adaptive immune responses once exposed to circulating immune cells.

Figure 2 depicts intracellular antigen processing following the administration of DNA or mRNA vaccines. DNA vaccines enter tumor and/or antigen-presenting host cells and translocate to the nucleus, where they are transcribed into mRNA that is exported to the cytoplasm. By contrast, mRNA vaccines remain in the cytoplasm and are directly translated by ribosomes [67,68]. Physical and chemical methods to improve DNA entry into APCs include electroporation, particle bombardment, microneedle arrays, and intranodal injection [69,70,71,72]. Key mRNA delivery platforms include lipid nanoparticles (LNPs), DC-targeted LNPs, and virus-like particles [73,74,75]. Both platforms result in the intracellular production of target proteins from DNA- or RNA-encoded sequences, which are processed into peptide fragments and routed through the endoplasmic reticulum and Golgi apparatus. Subsequently, these peptides are loaded onto MHC class I and II molecules and presented on the cell surface, initiating adaptive immune responses upon interaction with circulating T cells.

Table 1 summarizes the major vaccine delivery platforms, including antigen source, principal APC involvement, dominant antigen presentation pathways (MHC I vs. MHC II), degree of dendritic cell dependence, predominant T-cell response profiles (CD4^+^ vs. CD8^+^ bias), and the number of ongoing or completed Phase I–III clinical trials in our review. Although both humoral (B-cell) and cellular (T-cell) responses contribute to antitumor immunity, vaccine platforms can often be conceptualized according to CD4^+^- or CD8^+^-dominant response profiles [76]. Vector-based and nucleic acid vaccines may favor CD8^+^-biased cytotoxic responses through endogenous antigen expression and MHC class I presentation, whereas ex vivo APC-based vaccines more frequently generate balanced or CD4^+^-dominant responses due to direct antigen presentation and helper T-cell engagement [77,78,79,80,81,82,83,84,85,86].

Summary of vaccine types including platform name, antigen source, main antigen-presenting cells (APCs) involved, dominant antigen presentation pathways (MHC I vs. II), dendritic cell (DC) dependence, T-cell response profiles (CD4^+^ vs. CD8^+^), and the number of ongoing or completed clinical trials (Phases I/II/III). Vector-based and nucleic acid vaccines use CD8^+^ biased responses, while ex vivo APC-based vaccines elicit balanced or CD4^+^ dominant responses.

## 5. Vaccine Delivery Platform

Vaccine delivery platforms have been traditionally designed to deliver antigens without extensive modification to the tumor environment. Earlier cancer vaccine efforts around the new millennium focused on efficient antigen presentation through professional antigen-presenting cells (APCs), such as dendritic cells (DCs). Areas of active clinical investigation include synthetic long peptide (SLP) vaccines engineered for specific epitopes, viral vector (VV)-based platforms, and nucleic acid vaccines utilizing DNA or RNA constructs.

**Figure 3:**
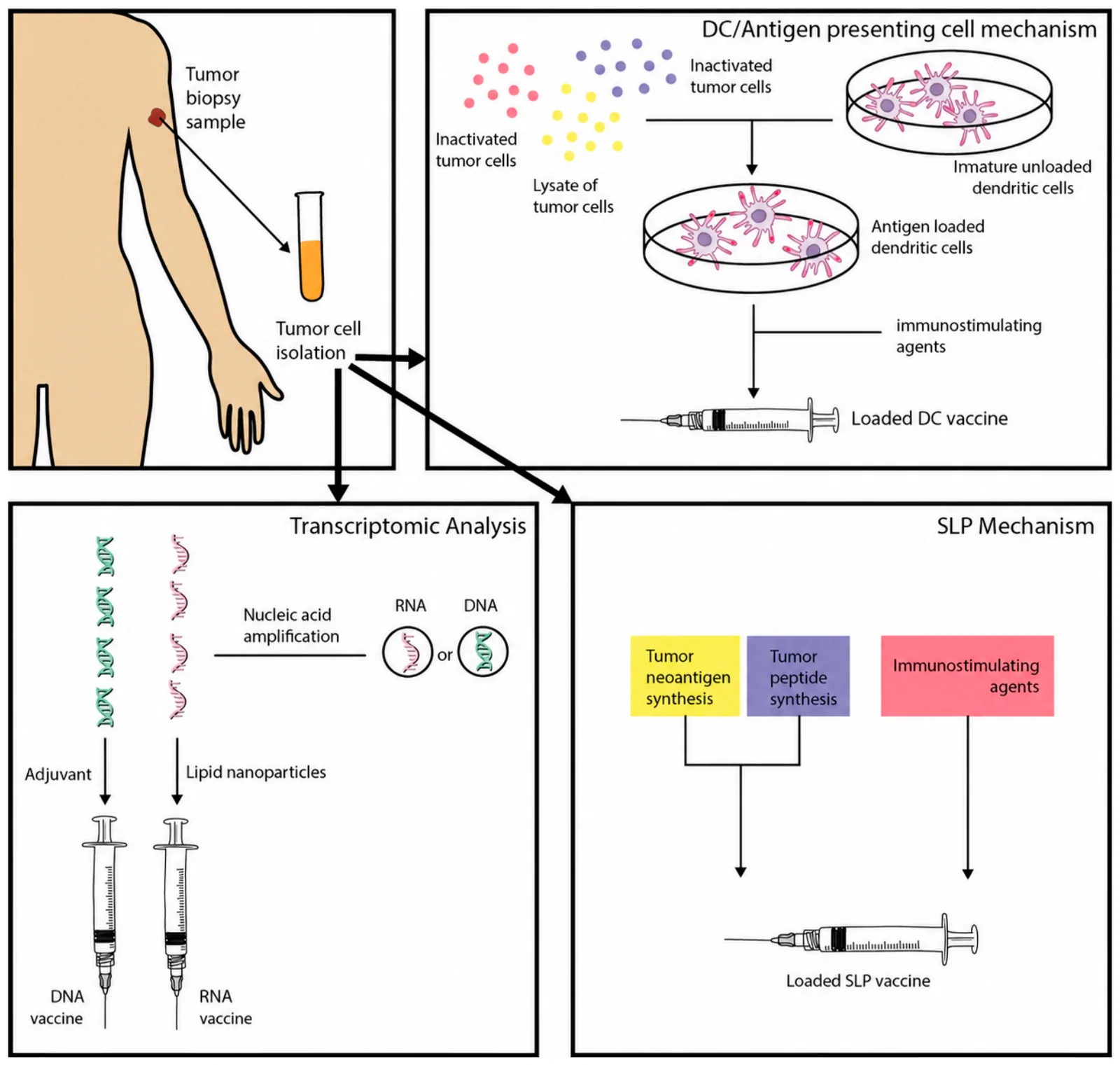
Production pathways for different types of melanoma vaccines. Schematic overview of how DC/APC, SLP, nucleic acid (DNA/mRNA) vaccines are produced. Arrows show pathways of vaccine platform production. Tumor tissue is either autologous or allogeneic and is processed via tumor cell isolation or proteomic or transcriptomic analysis. DC/APC vaccines (top right) are generated by loading DCs with inactivated tumor cells, tumor lysates, or extracellular vesicles, and then combined with immunostimulatory agents. SLP vaccines (bottom right) rely on identification and synthesis of tumor-specific peptides or neoantigens, which are formulated with adjuvants. Nucleic acid vaccines are produced by amplifying tumor-derived DNA or mRNA sequence encoding neoantigens, which are delivered with lipid nanoparticles (RNA) or adjuvants (DNA).

Figure 3 provides a schematic overview of how DC/APC, SLP, and nucleic acid (DNA/mRNA) vaccines are produced. Tumor tissue is either autologous or allogeneic and is processed via tumor cell isolation or proteomic or transcriptomic analysis [87,88,89]. DC/APC vaccines are generated by loading DCs with inactivated tumor cells, tumor lysates, or extracellular vesicles, and then combined with immunostimulatory adjuvants [90,91]. SLP vaccines rely on identification and synthesis of tumor-specific peptides or neoantigens, which are formulated with adjuvants [92]. Nucleic acid vaccines are produced by amplifying tumor-derived DNA or mRNA sequences encoding neoantigens, which are delivered via lipid nanoparticles (RNA-LNPs) or adjuvants (DNA) [93,94,95]. The “needle-to-needle” manufacturing time, spanning from biopsy to vaccination, for personalized cancer vaccines can vary widely between platforms, ranging from 1–3 days for tumor lysate platforms to 2–4 weeks for mRNA platforms [96,97].

Oncolytic viruses have also been investigated with ICIs to enhance antitumor immunity. Oncolytic viruses selectively infect and lyse tumor cells, inducing immunogenic cell death, releasing tumor antigens, and activating innate immune pathways that promote DC priming and CD8^+^ T-cell infiltration. Oncolytic viruses can convert immunologically “cold” tumors into “hot” tumors that are more responsive to checkpoint blockage [98].

Routes of administration include, but are not limited to, intravenous, intranodal, intradermal, subcutaneous, intramuscular, and intralymphatic routes [99].

Recent evidence in melanoma suggests that off-the-shelf SARS-CoV-2 mRNA–LNP vaccines sensitize tumors to immune checkpoint blockade and improve median and overall survival. mRNA vaccine platforms may exert intrinsic immunostimulatory adjuvant effects, blurring the traditional distinction between antigen delivery systems and adjuvants. However, the magnitude and clinical relevance of these effects in the context of cancer vaccination remains uncertain and are currently areas of active investigation [19]. The concurrent use of SARS-CoV2 vaccines could be an accessible and low-cost strategy for patients awaiting personalized neoantigen vaccines or in settings where individualized vaccines are not readily available.

## 6. Adjuvants in Melanoma Vaccines

A vaccine adjuvant enhances immunogenicity and is designed to activate the innate immunological response. In therapeutic melanoma vaccines, adjuvants can be broadly divided into five mechanistic classes, including (1) delivery/depot adjuvants; (2) pattern-recognition receptors (innate immunity) agonists; (3) cytokine and hematopoietic immune stimulators; (4) dendritic cell (DC) priming agents; and (5) immune microenvironment agents [100,101].

Delivery/depot adjuvants such as Montanide ISA 51VG and Freud’s adjuvants create a water-in-oil emulsion that sustains antigen release and promotes antigen-presenting cell (APC) uptake [102].

Pattern-recognition receptor agonists include poly-ICLC, CAF09b, and RBL001/RBL002 [103,104,105,106]. By mimicking pathogen-associated and damage-associated molecular patterns, these agonists activate innate immune pathways, such as toll-like receptor (TLR)-related signaling cascades, to trigger nuclear factor-kappa B (NF-κB)-inducible gene transcription and type I interferon responses [107].

Cytokine-based adjuvants include sargramostim (GM-CSF) and T-cell growth factors such as interleukin-2 (IL-2), which promote DC activation and activate cytotoxic T cells and NK cells, respectively [108,109].

Dendritic cell priming agents include CDX-301, an fms-like tyrosine kinase 3 (Flt3) ligand that expands DCs and hematopoietic progenitors to increase antigen presentation capacity [110]. Ex vivo loading of DCs may bypass the need for classical adjuvants, since DCs are matured in the lab rather than relying on in vivo recruitment via adjuvant-induced inflammation. However, ex vivo maturation does not fully recapitulate the quality of DC activation in vivo, as the most commonly used clinical maturation cocktail fails to produce IL-12p70, a key cytokine for Th1 polarization and CD8^+^ T-cell response [111].

Finally, immune modulates and tumor microenvironment agents modify immune suppression rather than stimulating immunity. Examples include Sirolimus (mTOR inhibitor), which modulates T-cell differentiation and memory formation, and cisplatinum (platinum based chemotherapy), which induces immunogenic cell death [112,113].

The majority of melanoma vaccine candidates in our analysis cohort use TLR agonists as adjuvants, either by themselves or conjugated to peptides and other particles. When selecting adjuvants for vaccine strategies, there are several key factors. These include the route of administration (e.g., intravenous, intramuscular, or intradermal), the desired immune polarization (CD4^+^, CD8^+^, or both), the delivery platform (e.g., peptide, protein subunit, DC–based, viral vector, or nucleic acid), and stage of disease (minimal residual vs. bulky metastatic burden) [114,115,116,117,118,119,120,121]. Host-related variables include age, ethnicity, baseline immune competence, comorbidities, prior therapies, and genetic background [122,123,124,125]. Finally, safety and tolerability must be weighed alongside production considerations such as scalability and cost [126,127,128,129]. These interdependent factors underscore that vaccine design is a tailored process requiring alignment between antigen presence, delivery platform, immunologic goals, and patient context.

## 7. Antigen Selection in Melanoma Vaccines

Over the past 15 years, melanoma vaccine clinical trials have shown a conceptual shift in antigen selection. Before 2010, melanoma vaccine research was dominated by tumor-associated antigens (TAAs), including synthesized peptides and those synthesized by allogeneic cells. After negative results from the DERMA, Canvaxin, and Schadendorf DC vaccine trials, there has been increasing adoption of tumor-specific antigens (TSAs). The greater adoption of TSAs has been driven by growth in next-generation sequencing techniques [130]. Both antigen strategies have shown encouraging clinical and immunologic signals, but they differ fundamentally in biology, production pipelines, and translational challenges.

### 7.1. Tumor-Associated Antigens

Tumor-associated antigens (TAAs) are antigens preferentially expressed in tumor cells compared to healthy tissue. In the clinical trials we examined, selected antigens included melanocyte lineage antigens, such as gp100, tyrosinase, tyrosinase-related proteins (TRP2), and MART-1, as well as cancer-testis antigens, such as MAGE-A3 and NY-ESO-1. Other shared antigens included hTERT (telomerase)- and PTEN (tumor suppressor)-related targets [131,132]. Several antigen-targeted DC vaccines employed multi-antigen loading strategies without disclosing individual TAAs.

Because TAAs are “shared” antigens across patients, they offer production scalability and broad applicability to appropriately selected patients [133]. However, key challenges involve their biologic overlap with normal tissues [134,135]. Many TAAs can be expressed in non-malignant cells, so the targeting of TAAs carries the risk of autoimmune-mediated toxicity [136,137]. The therapeutic window of TAAs also depends on sufficient enrichment in tumor tissues and minimal expression in normal tissue [138]. TAA-specific T cells may therefore be subject to central and peripheral tolerance, where high-affinity T cells are clonally deleted in the thymus or suppressed in the periphery [139,140].

Despite these biologic constraints, TAA-directed approaches continue to demonstrate clinical activity. BNT1111 (NCT04526899) is an active Phase II trial using 4 TAAs (NY-ESO-1, MAGE-A3, tyrosinase, and TPTE). As of February 2025, preliminary results show that the primary endpoint of improved Objective Response Rate (ORR) was met. Among 94 patients treated with BNT111 in combination with cemiplimab, the ORR was 18.1%, including 11 complete and 6 partial responses among 94 patients, exceeding the prespecified historic control ORR of 10%. Although BNT111 has a manageable safety profile, most secondary endpoints were not met. At a median 15.6 months of follow up, median progression-free survival (PFS) was 3.1 mo and median overall survival (OS) was 20.7 mo, with no meaningful differences observed between the vaccine combination arm and the cemiplimab comparator arm [141].

Outside of vaccine platforms, TAAs have been successfully targeted in other melanoma immunotherapy strategies. Tebentafusp (KIMMTRAK) is an FDA-approved prescription injection approved in 2022 for the treatment of HLA-A*02:01 positive (MHC I loci) metastatic or unresectable uveal melanoma [142]. Tebentafusp is a bispecific gp100-directed T-cell receptor fusion protein linked to a CD3 effector domain, which was shown to increase overall survival for patients with metastatic uveal melanoma (73% 1 yr OS in tebentafusp vs. 59% 1 yr OS in single-agent pembrolizumab, ipilimumab, or dacarbazine) [143].

### 7.2. Tumor-Specific Antigens/Neoantigens

Tumor-specific antigens (TSAs), commonly referred to as neoantigens, are epitopes encoded by tumor cells that arise from somatic mutations or, less frequently in melanoma, viral oncogene expression [144]. Melanoma has long been characterized by one of the higher tumor mutational burdens, with individual tumors harboring hundreds to thousands of mutations, creating a large number of potentially targetable neoepitopes [145,146,147]. Unlike TAAs, TSAs are not expressed in normal tissues and not subject to central immune tolerance, making them theoretically highly immunogenic [148].

Advances in next-generation sequencing and machine/deep learning-based antibody/antigen binding prediction algorithms have enabled the systematic identification, prediction, and prioritization of candidate neoantigens [149,150,151,152,153]. Concurrent neoantigen discovery efforts have revealed that the vast majority of TSAs are “private” neoantigens encoded by “passenger” mutations, which are unique to each individual’s tumors [154,155,156,157]. These findings have motivated ongoing efforts to test custom-made personalized vaccines.

However, biologic constraints remain. Few neoantigens are capable of producing a robust immune response [158]. The majority of aberrantly expressed TSAs (aeTSAs) arise from unstable short-lived proteins that are efficiently presented via direct MHC I presentation within tumor cells but are suboptimal substrates for cross-presentation by DCs [157,159]. Cancer cells are poor antigen-presenting cells, so the immune system depends on cross-presentation by dendritic cells (DCs) to detect the presence of TSAs. In established tumors, T-cell clones that initially respond to the most immunogenic antigens may already be exhausted and represent poor targets for reactivation by vaccination or checkpoint blockade, thus leading to immune checkpoints such as the PD-1/PD-L1 axis becoming the principal barrier to anti-tumor immunity. Neoantigen prediction algorithms that prioritize high immunogenicity may inadvertently select for antigens whose cognate T cells have undergone terminal exhaustion.

In the untreated host, most aeTSAs may remain undetected by the immune system; nonetheless, a small subset may provide attractive immunotherapy targets due to their high rates of expression and recurrence across many tumors [51,160]. Efforts are underway to find alternative sources of TSAs beyond canonical single-nucleotide-variant-derived neoantigens, including from frameshift mutations, gene fusions, aberrant splicing events, and translation from noncoding regions of the genome [161,162,163,164,165]. Moving forward, there is a need for standardization across sequencing platforms, mutation-calling pipelines, neoantigen-prediction algorithms, and immunogenicity assays [166,167,168]. Consortium efforts are underway to harmonize these methodologies [169,170,171].

TSA-directed vaccine strategies are actively being evaluated with mixed but evolving clinical signals. EVX-01 (NeoPepVac; NCT05309421) is an ongoing open-label, single-arm Phase II proof-of-concept trial evaluating the EVX-01 vaccine in combination with pembrolizumab in checkpoint inhibitor-naïve patients with unresectable or metastatic melanoma. The primary objective is to determine whether EVX-01 can improve the best overall response in patients who achieve initial stable disease or partial response to pembrolizumab [172]. Preliminary data suggest EVX-01 is well-tolerated and induces neoantigen-specific immune responses [173].

Not all neoantigen-based approaches have demonstrated benefit. The Phase II INITIUM trial evaluating the UV1 vaccine (NCT04382664) in combination with nivolumab and ipilimumab enrolled 156 patients and did not meet its primary endpoint of improved PFS. At a minimum follow-up of 18 months, median PFS was improved in the vaccine arm compared to dual therapy (HR 0.95). No significant differences were observed in OS or ORR between the vaccine and dual therapy arms [174,175].

More encouraging results have emerged from the Phase II KEYNOTE-942 trial (NCT05933577), which evaluated personalized mRNA-4157 (now known as intismeran autogene) in combination with pembrolizumab in patients with resected high-risk melanoma. The addition of the neoantigen vaccine significantly improved recurrence-free survival (HR 0.51) and distant metastasis-free survival (HR 0.38) compared to pembrolizumab monotherapy, with a manageable safety profile [176]. With a median follow-up of approximately three years, the benefit has remained durable [20,177]. The most recent analysis suggests a 49% reduction in the risk of recurrence or death at 3 and 5 years [20,21,178]. A Phase III trial is currently ongoing, representing one of the most advanced evaluations of personalized neoantigen vaccination in melanoma [179].

### 7.3. Whole-Tumor Lysate

Whole-tumor and tumor lysate vaccines represent an antigen agnostic strategy that does not fit neatly within TAA- or TSA-directed frameworks. These platforms include tumor lysate DC vaccines and autologous tumor RNA approaches that do not preselect an antigen class, instead exposing the immune system to the full repertoire of tumor-derived proteins [180]. Incorporation of TAAs and TSAs may potentially broaden immune coverage and limit antigen escape.

Earlier clinical experience with whole-tumor vaccines, like many therapeutic cancer vaccines, was tempered by poor immunogenicity and the suppressive influence of the tumor microenvironment [181,182,183]. However, these shortcomings may reflect the immunologic context of the time rather than inherent limitations of whole-tumor antigens. Recent preclinical evidence in mouse models suggest that neoantigens predicted on the sole basis of in silico peptide–MHC binding affinity underperform relative to whole-tumor lysate vaccines. Instead, effective in vitro peptide–MHC binding affinity and peptide immunogenicity significantly improve the prioritization of tumor-rejecting neoepitopes [184,185]. Experimentally validated HLA binding may outperform prediction-based selection alone, underscoring the potential value of broader antigen presentation. Manufacturing considerations also differ substantially, as tumor lysate vaccines can be produced within 1–3 days from resected tissue, compared to approximately nine weeks required for individualized mRNA vaccine production [186].

Clinically, the Phase II TLPLDC/TLPO trial (NCT02301611) evaluated autologous tumor lysate vaccines in resected stage III/IV melanoma. However, the trial had substantial protocol modifications that complicate interpretation. The initial design was a randomized, double-blind placebo-controlled trial comparing the dendritic cell vaccine (TLPLDC) with a placebo (2:1 randomization, *n* = 124), before being amended to replace the placebo arm with the tumor lysate particle-only (TLPO) platform, enrolling 63 additional patients with the resulting allocation (placebo *n* = 41, TLPLDC *n* = 103, TLPO *n* = 43). A major confound was the non-randomized use of G-CSF before dendritic cell collection. Patients receiving G-CSF had worse outcomes than those without G-CSF (36-month DFS 24.4% vs. 55.8%; OS 69.8% vs. 94.2%). Outcomes in the TLPLDC + G-CSF group were similar to placebo, which suggests G-CSF pretreatment may have abrogated vaccine efficacy [187]. The study did not stratify patients by use of standard-of-care immune checkpoint inhibitors, and it is notable that standard-of-care systemic therapy during the study period transitioned from chemotherapy and interferon-α to immune checkpoint inhibitors (ICIs). Although disease-free survival (DFS) did not differ in the intention-to-treat analysis, per-treatment analysis showed improved 24-month DFS in vaccinated patients who received prior or concurrent checkpoint inhibitor therapy (62.9% vs. 34.8%) [188]. Among patients with resected recurrent disease, 24-month DFS was 88.9% versus 33.3% (*p* = 0.013) in per-treatment analysis. Over 36 months, the tumor lysate strategy was associated with improved DFS (HR 0.52, *p* < 0.01) [189]. A notable weakness of the TLPLDC and TLPO trials in comparison to mRNA-4157/intismeran autogene is that only 37% of patients received standard-of-care ICI monotherapy. Among vaccinated patients who did not receive ICI, there was no significant difference in 36-month DFS (*p* = 0.184), although an overall survival benefit was observed. In 2024, the parent company Elios Therapeutics announced plans to initiate a Phase III trial; as of July 2026, the trial has yet to be registered on ClinicalTrials.gov [190].

## 8. A Critical Assessment of Peer-Reviewed Publication of Randomized Trials

Comparing clinical trial outcomes of melanoma vaccines is challenging due to heterogeneity in primary and secondary endpoints, including differing definitions of progression, clinicopathologic outcomes, and survival metrics. Additional complexity arises from evolving standard-of-care designs and multiple drug or platform name changes across trial phases. In many vaccine trials, comparator arms still rely on monotherapy checkpoint inhibition, which may not reflect evidence-based approaches supporting the use of dual-checkpoint inhibitors [191,192]. Inadequate and poor-quality outcome reporting in clinical trials is a well-documented problem that impedes the ability of researchers to evaluate, replicate, synthesize, and build upon study findings in melanoma vaccine development [193].

Publication bias lao favors positive results and hinders comprehensive review. There is little incentive for companies sponsoring trials to publish negative results promptly, in full, or publish any results at all. For example, the DERMA trial was completed in 2011, but its results were not published until 2018. Similarly, the Phase III POL-103A (Seviprotimut-L) trial was terminated early in 2021, with limited results made publicly available as of 2026.

To promote harmonized and transparent reporting of outcomes in trial protocols and published reports, initiatives such as the Instrument for Reporting Planned Endpoints in Clinical Trials (InsPECT) have been developed [194]. The final product will provide unique InsPECT extensions to the SPIRIT (Standard Protocol Items: Recommendations for Interventional Trials) and CONSORT (Consolidated Standards of Reporting Trials) reporting guidelines [195]. Vaccine researchers, sponsoring institutions, and regulatory bodies should consider implementing these toolkits to improve the reproducibility and comparability of future clinical trials.

## 9. Summary and Challenges of Key Clinical Trials

Table 2 includes a summary of vaccine names, sponsoring institutions, drug platforms, clinical trial phases, recruitment status, trial identifiers (NCT numbers), study completion dates, concurrent therapies, and key therapies that have shaped design rationale for therapeutic melanoma vaccines. A total of 14 unique vaccine candidates were identified across 15 trials (defined as stage II or higher, completed from 2005 to 2025, with publicly available results).

Four vaccine candidates reached Phase III large-scale efficacy trials, including one dendritic cell vaccine (nDC), one synthetic long peptide vaccine (Cylembio), one polyvalent vaccine (POL-103A/Seviprotimut-L), and one mRNA-based (intismeran autogene) vaccine. One mRNA trial is currently active.

The Phase III MIND-DC trial of the dendritic cell vaccine nDC (NCT02993315) evaluated a personalized DC-based platform designed to induce tumor-specific immune responses. Patients were randomized to receive the vaccine or placebo. The primary endpoint was 2-year recurrence-free survival (RFS), with secondary endpoints including median RFS, OS, safety, and immunologic response. The 2-year RFS rate was 36.8% in the nDC group compared to 46.9% in the control group (*p* = 0.31). The median RFS was 12.7 months versus 19.9 months (hazard ratio [HR] 1.25; 90% CI 0.88–1.79; *p* = 0.29). Median OS was not reached in either group. Despite the lack of clinical benefit, functional antigen-specific T-cell responses were detected in 67.1% of patients in the treatment arm compared to 3.8% in the control group (*p* < 0.001), demonstrating immunogenicity without a corresponding survival advantage [196]. The authors proposed several potential explanations for the trial’s lack of efficacy. These include changes in standard of care with anti-PD1 that limited accrual; variability in the gut microbiome, which may confer immunotherapy resistance; and factors related to the magnitude of immunological response, cytotoxic capacity of induced T cells, and choice of tumor antigens [197,198]. The authors noted that neoantigens could be more immunogenic and a better target for future vaccine development [144].

Despite the negative Phase III signal from MIND DC, its findings do not provide definitive evidence against the use of dendritic cell vaccines as anti-cancer therapeutic vaccines. Earlier randomized Phase II data demonstrated that DC-mediated antigen presentation is superior to irradiated tumor cell vaccines, with a 70% reduction in risk of death (HR 0.304; *p* = 0.005) and a median OS of 43.4 versus 20.5 months at 5-year follow-up [199,200]. Similarly, the TriMixDC-MEL randomized Phase II trial demonstrated that engineering DC activation through mRNA electroporation with CD40L, CD70, and constitutively activated TLR4 improved the 1-year DFS to 71% versus 35% (*p* = 0.044) in resected stage III/IV melanoma [201]. These earlier positive results suggest that the MIND-DC failure may reflect limitations specific to the nDC platform rather than DC-based approaches as a category.

Keynote D18 was a Phase III trial evaluating Cylembio (AKA IO102/IO103 AKA imsapepimut/etimupepimut; NCT05155254) for advanced melanoma. Patients received Cylembio with pembrolizumab or pembrolizumab alone. Cylembio is an SLP vaccine designed as an immune modulator to target indoleamine 2,3-dioxygenase (IDO)- and PD-L1-expressing cells, thereby enhancing immunogenicity. Topline results from August 2025 showed an improved median PFS to 19.4 months vs. 11 months for monotherapy (HR 0.77; 95% CI 0.58–1.00; *p* = 0.0558), narrowly missing the prespecified threshold for statistical significance. Exploratory analyses suggested greater benefit among patients with PD-L1-negative tumors (HR 0.54; 95% CI 0.35–0.85), a high risk with historically poor response to pembrolizumab. Overall survival data were expected in early 2026 to clarify the long-term clinical benefit of the Cylembio regimen. However, by late 2025, the parent company IO Biotech had laid off half its workforce and was exploring ‘strategic alternatives’, making the release of mature overall survival data uncertain [202,203].

The MAVIS trial of POL-103A (NCT01546571) was a Phase III study granted fast track designation, terminated after interim analysis, and then reportedly redesigned for a pivotal trial. POL-103A (AKA Seviprotimut-L) is an allogenic tumor lysate-based polyvalent vaccine designed to prevent recurrence in high-risk post resection (Stage IIB, IIC, or III) melanoma patients. The primary endpoint of recurrence-free survival (RFS) was not met, with an overall hazard ratio of 0.881 (*p* = 0.46). However, exploratory subgroup analyses revealed heterogeneous effects across disease stages. In stage IIB/IIC patients, the HR was 0.67 (95% CI: 0.37–1.19), with particularly strong signals in patients younger than 60 years (HR = 0.324, 95% CI: 0.121–0.864) and those with ulcerated primary melanomas (HR = 0.493, 95% CI: 0.255–0.952). The investigators concluded POL-103A warrants further study in younger patients and those with ulcerated melanomas. In October 2025, the vaccine technology and parent company PolyNoma LLC was acquired for $165 million. It is unknown if the positive signals in the younger and ulcerated melanoma subset will translate into future confirmatory trials.

Finally, the ongoing Phase III INTerpath-001 trial (NCT05933577) is evaluating the personalized intismeran autogene mRNA vaccine (aka mRNA-4157/V940) in combination with pembrolizumab in patients with resected high-risk melanoma. The addition of the neoantigen vaccine significantly improved recurrence-free survival (HR 0.51) and distant metastasis-free survival (HR 0.38) compared to pembrolizumab monotherapy, with a manageable safety profile [176]. The most recent analysis suggests a 49% reduction in the risk of recurrence or death at 3 and 5 years [20,21]. Final readout is expected in 2029 once sufficient endpoints have been met for planned statistical analysis.

## 10. Future Directions

In summary, the major advances in our understanding of the rationale for therapeutic cancer vaccines can be categorized into four interconnected concepts. These principles will guide melanoma vaccine development work for the foreseeable future.

First, the clinical efficacy of immune checkpoint inhibitors, beginning with ipilimumab in metastatic melanoma and then across multiple tumor types, confirmed the existence of cancer immune surveillance based on recognition of autologous non-self neoantigens, thus validating that the immune system can be used to eliminate established tumors [34].

Second, advances in genomic sequencing and immunogenomics suggest that patient-specific neoantigens rather than shared tumor-associated antigens or driver mutations are the primary targets of endogenous anti-tumor immunity [204].

Third, clinical trials have confirmed that therapeutic cancer vaccines can induce and enhance anti-tumor immune responses as monotherapies; however, just as in natural anti-tumor immunity, these vaccine-induced responses are limited by immune checkpoint-mediated suppression. Optimal clinical benefit requires combination with checkpoint blockade, as demonstrated by the positive results of the KEYNOTE-942/V940 trial and the negative results of DC vaccine monotherapy in the Phase III MIND-DC trial, despite robust immunogenicity [21,196].

Fourth, patients may exhibit primary resistance due to immunologically “cold” tumors without sufficient T-cell infiltration, while others develop acquired resistance after initially responding to therapy. Oncolytic viruses such as T-VEC suggest cold-to-hot conversion is possible, and therapeutic vaccines may have a complementary role in converting cold tumors into hot tumors through de novo T-cell priming and broadening of the neoantigen-specific T-cell repertoire [205].

## 11. Conclusions

The therapeutic landscape for advanced melanoma has undergone a significant shift in the past 15 years toward autologous neoantigens and co-stimulatory molecules. Despite the advances in immunotherapy and targeted therapies that have revolutionized the treatment paradigm for melanoma, there is a persistent need to develop therapies, particularly for patients who have progressed on ICIs or for those patients who are poor candidates for existing immune-based therapies. The rational design of therapeutic vaccine candidates and their combination with targeted therapies will be a critical opportunity to continue optimizing disease control for melanoma.

## Figures and Tables

**Table 1:** Mechanistic overview of melanoma vaccine platforms.

Vaccine Type	Antigen Source	Primary APC Involved	Presentation Pathway	DC-Dependent?	CD4/CD8 Response?	No. of Trials (Phase I/II/III)
Dendritic Cell *	Antigen-loaded DCs (ex vivo)	Injected DCs	MHC I & II (ex vivo priming)	Yes	CD4^+^ & CD8^+^	6/7/2
Tumor Lysate ^	Tumor lysate or peptide-loaded APCs	DCs, macrophages,	MHC II (some cross-presentation)	Yes	CD4^+^ >> CD8^+^	3/3/3
Synthetic Long Peptide	Long peptides spanning T-cell epitopes	Endogenous APCs (esp. DCs)	MHC II → CD4^+^; MHC I via cross-presentation	Yes	CD4^+^ & CD8^+^ (if cross-presented)	16/14/4
Viral Vector	DNA encoding an antigen via viral delivery	Infected cells + APCs	MHC I (infected cells); MHC II (cross-presentation)	Indirect	CD8^+^ > CD4^+^	2/0/1
mRNA	mRNA encoding an antigen	Transfected cells & APCs	MHC I (endogenous), MHC II (via uptake)	Indirect	CD4^+^ & CD8^+^	5/3/1
DNA	Plasmid encoding an antigen	Muscle cells, DCs (if taken up)	MHC I (mainly), MHC II (if secreted)	Indirect	CD8^+^ > CD4^+^	1/3/0

* DCs are considered to be the most effective APCs for loading and presenting antigens for vaccines. ^ Tumor cell lysate can be injected as a vaccine or incubated with DCs for antigen loading.

**Table 2:** Summary of noteworthy melanoma vaccine clinical trials (2005–2025).

Vax Name	Institution	Platform	Number of Targets	Trial ID	Phase	Status	Completion	Additional Therapies	Takeaway
CancerVax (Canvaxin)	CancerVax Corp/John Wayne Cancer Institute	Tumor Lysate	3 melanoma cell lines	NCT00052156 (Stage IV); NCT00052130 (Stage III)	3	Terminated	04-2005	None	Failed, no DFS or OS benefit
Schadendorf DC	DeCOG	DC	MHC class I and II peptides	pre-registry era	3	Completed	~2005	None (vs. dacarbazine)	Failed. First Phase III DC vaccine trial in melanoma. ORR similarly low (DC 3.8% vs. DTIC 5.5%). Closed at interim analysis.
ECOG 1696	ECOG	SLP	3 peptides (gp100, MART-1, tyrosinase)	pre-registry era	2	Completed	~2006	IFN-α2b, GM-CSF, or both	Mixed. Immune responders had longer OS (21.3 vs. 13.4 mo; *p* = 0.046), suggesting immunologic response correlates with benefit.
EORTC 18961	EORTC	TAA	1 Ag (GM2)	NCT00005052	3	Terminated	~2009	None (vs. observation)	Failed. No RFS benefit (HR 1.00); detrimental OS trend (HR 1.66; *p* = 0.02). Stopped at second interim analysis.
gp100	NCI	SLP	1 peptide (HLA-A2)	NCT00019682 (with IL-2); NCT00094653 (with ipilimumab)	3	Completed	2011	High-dose IL-2; Ipilimumab	Mixed: With IL-2: improved ORR (16% vs. 6%; *p* = 0.03) and PFS. As comparator in ipilimumab trial: gp100 alone inferior (OS 6.4 mo), but helped establish ipilimumab’s efficacy.
DERMA trial	GlaxoSmithKline	SLP	1 Ag (MAGE-A3)	NCT00796445	3	Terminated	09-2015	None	Failed. Largest adjuvant melanoma vaccine trial (*n* = 1345). No DFS benefit (HR 1.01; *p* = 0.86) or OS benefit (HR 1.07) despite immunogenicity.
POL-103A (Seviprotimut-L)	Polynoma LLC	Tumor lysate	Unspecified	NCT01546571	3	Terminated	06-2021		Failed. Phase III terminated; no efficacy signal reported.
nDC (MIND-DC)	Radboud University Medical Center	DC (autologous CD1c+ cDC and pDC)	2 Ag (gp100, tyrosinase)	NCT02993315	3	Completed	2022	None (vs. placebo)	Failed. No RFS benefit (2-yr RFS 36.8% vs. 46.9% placebo). Strong immunogenicity (67.1% vs. 3.8%) without clinical benefit.
TLPLDC	Elios Therapeutics, LLC	APC		NCT02301611	2	Completed	05-2022	SoC Adjuvant Therapy	Mixed. ITT analysis negative for DFS. Interpretation limited by non-randomized ICI use and protocol amendments.
UV1	Zelluna Immunotherapy	SLP	3 peptides (hTERT)	NCT04382664	2	Completed	04-2024	Nivolumab, Ipilimumab	Phase II combination results pending full publication.
EVX-01 (NeoPepVac)	Evaxion Biotech	SLP	5–10 NeoAgs	NCT05309421	2	Active, Not Recruiting	07-2025	Pembrolizumab	Pending results with pembrolizumab combination.
TLPO	Elios Therapeutics, LLC	APC		NCT06175221	2	Active, Not Recruiting	06-2027	SoC Adjuvant Therapy	Ongoing. Phase III announced but not yet registered as of 03-2026.
Cylembio (IO102/IO103)	IO Biotech	SLP	2 peptides (IDO1 and PDL1)	NCT05155254	3	Active, Not Recruiting	09-2027	Pembrolizumab	Failed. PFS numerically favored combination (19.4 vs. 11.0 mo; HR 0.77) but missed statistical significance (*p* = 0.056).
Intismeran autogene (mRNA-4157/V940)	Moderna/Merck	mRNA	Up to 34 NeoAgs	NCT03897881	2	Active, Not Recruiting	09-2029	Pembrolizumab	Promising. KEYNOTE-942: 44% reduction in recurrence/death (HR 0.561; *p* = 0.0266) with V940 + pembrolizumab vs. pembrolizumab alone in resected high-risk melanoma. First positive Phase II trial for neoantigen vaccine + ICI.
				NCT05155254	3	Active, Not Recruiting	09-2027	Pembrolizumab	Phase III ongoing in first-line unresectable/metastatic melanoma with pembrolizumab.

Table includes vaccine names, sponsoring institutions, drug platforms, clinical trial phases, recruitment status, trial identifiers (NCT numbers), study start/end dates, adjuvants, and concurrent therapies. A total of 14 unique vaccine candidates were identified across 15 trials (defined as stage II or higher with publicly available results) that have shaped design rationale for therapeutic melanoma vaccine. One synthetic long peptide (Cylembio/IO102/IO103), one tumor lysate (POL-103A/Seviprotimut-L), and one dendritic cell vaccine candidate failed in Phase III (nDC). One mRNA (intismeran autogene/mRNA-4157/v940) vaccine candidate is currently in Phase III trials. Table info is up to date as of 28 July 2026.

## Data Availability

Research data available upon email request to corresponding authors.

## Supplementary Materials

The following supporting information can be downloaded at: https://www.mdpi.com/article/10.3390/vaccines14080682/s1, Section S1. Clinical Registry Search Strategy. A systematic search of the ClinicalTrials.gov registry database was conducted to identify therapeutic cancer vaccine trials evaluated in patients with cutaneous and advanced melanoma. Search criteria are listed below. Table S1. Two-Tier Screening Eligibility Matrix. Studies identified from ClinicalTrials.gov underwent a two-tier screening process using predefined inclusion and exclusion criteria. Figure S1. PRISMA 2020 Flow Diagram, Figure S1 illustrates the systematic selection process following PRISMA 2020 guidelines for study registers and trial databases, with snowballing of relevant trials.

## Abbreviations

The following abbreviations are used in this manuscript:

aeTSAs	Aberrantly Expressed Tumor-Specific Antigens
Ag/Ags	Antigen/Antigens
AI	Artificial Intelligence
AJCC8	American Joint Committee on Cancer (8th Edition)
APC/APCs	Antigen-Presenting Cell/Cells
CD/CD4/CD8/CD70	Cluster of Differentiation (e.g., CD4^+^ T cell, CD8^+^ cytotoxic T cell, CD70)
cDC	Conventional Dendritic Cell
CD40L	CD40 Ligand
CI	Confidence Interval
CONSORT	Consolidated Standards of Reporting Trials
DC/DCs	Dendritic Cell/Cells
DeCOG	Dermatologic Cooperative Oncology Group
DFS	Disease-Free Survival
DNA	Deoxyribonucleic Acid
DTIC	Dacarbazine
ECOG	Eastern Cooperative Oncology Group
EORTC	European Organisation for Research and Treatment of Cancer
FDA	Food and Drug Administration
Flt3	Fms-like Tyrosine Kinase 3
G-CSF	Granulocyte Colony-Stimulating Factor
GM-CSF	Granulocyte-Macrophage Colony-Stimulating Factor
GM2	Ganglioside M2
gp100	Glycoprotein 100
HLA	Human Leukocyte Antigen
HR	Hazard Ratio
hTERT	Human Telomerase Reverse Transcriptase
ICI/ICIs	Immune Checkpoint Inhibitor/Inhibitors
IDO/IDO1	Indoleamine 2,3-dioxygenase/Indoleamine 2,3-dioxygenase 1
IFN-α/IFN-α2b	Interferon-alpha/Interferon-alpha 2b
IL-2/IL-12p70	Interleukin-2/Interleukin-12 p70
InsPECT	Instrument for reporting Planned Endpoints in Clinical Trials
ITT	Intention-To-Treat
LNP/LNPs	Lipid Nanoparticle/Nanoparticles
MAGE-A3	Melanoma Antigen Gene E-A3
MART-1	Melanoma Antigen Recognized by T-cells 1
MHC	Major Histocompatibility Complex
mRNA	Messenger Ribonucleic Acid
mTOR	Mammalian Target of Rapamycin
NCI	National Cancer Institute
NCT	National Clinical Trial (ClinicalTrials.gov registry identifier prefix)
nDC	Natural Dendritic Cell (or specific candidate name designation)
NeoAgs	Neoantigens
NF-κB	Nuclear Factor Kappa B
NK	Natural Killer (cells)
NY-ESO-1	New York Esophageal Squamous Cell Carcinoma 1
ORR	Objective Response Rate
OS	Overall Survival
pDC	Plasmacytoid Dendritic Cell
PD-1/PD1	Programmed Cell Death Protein 1
PD-L1	Programmed Death-Ligand 1
PFS	Progression-Free Survival
PhD	Doctor of Philosophy
PRISMA	Preferred Reporting Items for Systematic Reviews and Meta-Analyses
PTEN	Phosphatase and Tensin Homolog
RFS	Recurrence-Free Survival
RNA	Ribonucleic Acid
SARS-CoV-2	Severe Acute Respiratory Syndrome Coronavirus 2
SLP/SLPs	Synthetic Long Peptide/Peptides
SoC	Standard of Care
SPIRIT	Standard Protocol Items: Recommendations for Interventional Trials
TAA/TAAs	Tumor-Associated Antigen/Antigens
T-VEC	Talimogene Laherparepvec
Th1	T helper type 1
TLR/TLR4	Toll-Like Receptor/Toll-Like Receptor 4
TLPLDC	Tumor Lysate Particle Loaded Dendritic Cell
TLPO	Tumor Lysate Particle-Only
TPTE	Transmembrane Phosphatase with Tensin Homology
TRP2	Tyrosinase-Related Protein 2
TSA/TSAs	Tumor-Specific Antigen/Antigens
VV	Viral Vector
