# Supplementary material for: Advances in Therapeutic Melanoma Vaccines (2010–2025)
Source: Vaccines (Basel). 2026 Aug 7;14(8):682. doi: 10.3390/vaccines14080682 (PMC13517315; doi:10.3390/vaccines14080682)

Supplementary Methods

Section S1. Clinical Registry Search Strategy

A systematic search of the ClinicalTrials.gov registry database was conducted to identify therapeutic cancer vaccine trials evaluated in patients with cutaneous and advanced melanoma. Search Criteria are listed below.

- **Database / Search Registry:** ClinicalTrials.gov
- **Search Execution Date Range:** Trials registered or started between **June 01, 2010** and **February 01, 2025**.
- **Search Engine Parameters:**
  - **Condition / Disease Terms:** melanoma OR Melanomas OR Malignant Melanoma OR cutaneous melanoma OR advanced melanoma OR Melanoma (Skin)
  - **Intervention Terms:** vaccine OR vaccination OR immunization OR vaccinate OR immunize
  - **Start Date Constraint:** 2010-06-01_2025-02-01
  - **Sorting Parameter:** StudyFirstPostDate
- **Direct Query URL:** https://clinicaltrials.gov/search?cond=melanoma%20OR%20Melanomas%20OR%20Malignant%20Melanoma%20OR%20cutaneous%20melanoma%20OR%20advanced%20melanoma%20OR%20Melanoma%20(Skin)&intr=vaccine%20OR%20vaccination%20OR%20immunization%20OR%20vaccinate%20OR%20immunize&start=2010-06-01_2025-02-01&sort=StudyFirstPostDate
- **Total Initial Identified Records:** $N = 120$ studies

- **Clinical trial status extracted from ClinicalTrials.**gov Study Records

Table S1. Two-Tier Screening Eligibility Matrix

Studies identified from ClinicalTrials.gov underwent a two-tier screening process using predefined inclusion and exclusion criteria.

Screening Stage	Inclusion Criteria	Exclusion Criteria & Excluded Interventions

Stage 1: Title & Abstract Screening	• Target Disease: Diagnosis of cutaneous, advanced, resected high-risk, or metastatic melanoma.<br><br>• Intervention Type: Active vaccine component designed to elicit target antigen-specific immune response.<br><br>• Study Start Date: Trial start date between June 01, 2010 and February 01, 2025.	• Wrong Intervention Type: Immunotherapy drugs/biologics lacking an antigen-specific vaccine design.<br><br>• Passive Adjuvants Only: Evaluation of adjuvants in isolation (e.g., Poly-ICLC or GM-CSF monotherapy).<br><br>• Non-Vaccine Primary Focus: Trials evaluating external modalities where the vaccine is non-primary (e.g., SBRT radiotherapy trials).<br><br>• Off-Target Infectious Vaccines: Prophylactic infectious disease vaccines (e.g., smallpox/vaccinia immunization).
Stage 2: Full-Text / Registry Reporting Review	• Data Availability: Clinical trials with peer-reviewed published outcomes or completed registry reporting.<br><br>• Active/Ongoing Phase III: Ongoing pivotal trials with official interim updates.<br><br>• Valid Vaccine Mechanism: Verified peptide, nucleic acid (mRNA/DNA), dendritic cell, viral vector, or tumor lysate platform.	• Trial Terminated Without Data: Terminated, withdrawn, or suspended trials lacking publicly available or reported data.<br><br>• No Results Reported: Completed trials with no peer-reviewed publication or posted registry data.<br><br>• Wrong Intervention Class: Cellular therapies or non-vaccine drug immunotherapies (e.g., IL-15 cytokines, OBX-115 TIL cell therapy).<br><br>• Out-of-Bounds Start Date: Trials with true start dates prior to June 01, 2010.

Figure S1. PRISMA 2020 Flow Diagram

Figure S1 illustrates the systematic selection process following PRISMA 2020 guidelines for study registers and trial databases, with snowballing of relevant trials.

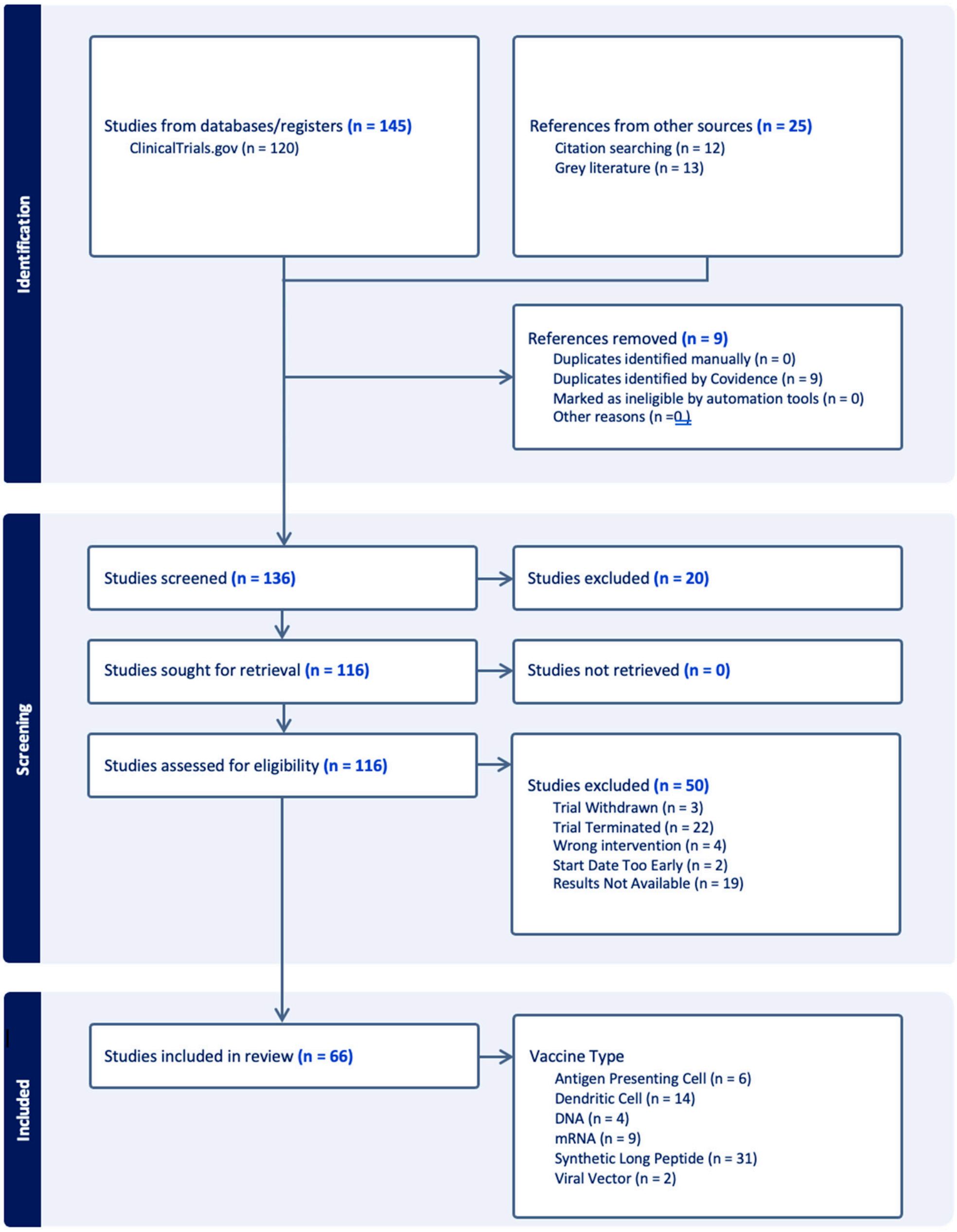

Supplement: Supplementary file 1 [file vaccines-14-00682-s001.zip › vaccines-4423792-supplementary.pdf]
